# Supplementary material for: Combination of Transcriptomics and Proteomics Reveals Differentially Expressed Genes and Proteins in the Skin of EDAR Gene-Targeted and Wildtype Cashmere Goats
Source: Animals (Basel). 2023 Apr 24;13(9):1452. doi: 10.3390/ani13091452 (PMC10177055; doi:10.3390/ani13091452)
Supplement: Supplementary file 1 [file animals-13-01452-s001.zip › Supplementary Table S1.pdf]

Supplementary Table S1 Primer information for RT- qPCR

Gene	Primer sequence
GHRHR	CCTGACCTGCCTCTTAGCCT
	TTGATGATCCACCAGTAGGG
IL36B	TCTGGGTGGCTGAATCCTGT
	ATCCATGTACGGTGGTCTC
JAKMIP1	TTGCGGTCCAGAAGACATCG
	CCTCTTCTCGGGTTGTTGTAT
JSRP1	GGACACCAAGCCCAAGAAGATG
	TGGTCTGTGCCTTCTTCCTTGC
MAP34-B	GCCGTGCTTCGTGCTGTGGAT
	CACTGTTTCACCAGCCCATTCT
MOGAT1	AGGTGGAAACATCTCAATCA
	ACTGGCACCAAATAAGCACC
PAEP	AGAAATGGGAGAACGGTGAGTG
	CTCGGGCTCAGCACTGTTTT
TYRP1	AAGAACACCTGCGACATTTGC
	CCCAGGGTATCATAATCTTC
