# Supplementary material for: Combination of Transcriptomics and Proteomics Reveals Differentially Expressed Genes and Proteins in the Skin of EDAR Gene-Targeted and Wildtype Cashmere Goats
Source: Animals (Basel). 2023 Apr 24;13(9):1452. doi: 10.3390/ani13091452 (PMC10177055; doi:10.3390/ani13091452)
Supplement: Supplementary file 1 [file animals-13-01452-s001.zip › Supplementary Table S2 .pdf]

Supplementary Table S2 Summary of clean data for transcriptome

Sample	WT01	WT02	WT03	EDAR01	EDAR02	EDAR03
Raw Reads Number	49,238,848	46,677,394	46,789,616	49,177,544	45,543,572	47,612,648
Raw Bases Number	7,385,827,200	7,001,609,100	7,018,442,400	7,376,631,600	6,831,535,800	7,141,897,200
Clean Reads Number	47,459,130	45,578,664	45,630,714	47,835,374	44,430,710	46,326,914
Clean Reads Rate(%)	96.39	97.65	97.52	97.27	97.56	97.3
Clean Bases Number	7,118,869,500	6,836,799,600	6,844,607,100	7,175,306,100	6,664,606,500	6,949,037,100
Raw Q30 Bases Rate(%)	92.77	91.72	91.75	91.4	92.49	90.91
Clean Q30 Bases Rate(%)	93.22	92.51	92.59	92.27	93.29	91.86
